# The effects of depression, anxiety and stress symptoms on the clinical pregnancy rate in women undergoing IVF treatment

**DOI:** 10.1186/s13104-019-4294-0

**Published:** 2019-05-09

**Authors:** Saman Maroufizadeh, Behnaz Navid, Reza Omani-Samani, Payam Amini

**Affiliations:** 1 School of Nursing and Midwifery, Guilan University of Medical Sciences, Rasht, Iran; 2 Department of Epidemiology and Reproductive Health, Reproductive Epidemiology Research Center, Royan Institute for Reproductive Biomedicine, ACECR, P.O. Box: 16635-148, Tehran, Iran; 3 Department of Biostatistics and Epidemiology, School of Public Health, Ahvaz Jundishapur University of Medical Sciences, Ahvaz, Iran

**Keywords:** Depression, Anxiety, Stress, Clinical pregnancy, IVF

## Abstract

**Objective:**

Women undergoing assisted reproductive technology treatment, are often anxious and depressed because of their fertility problem and the uncertainties of the treatment with which they have to deal. On the other hand, recent studies have shown that the effects of psychological distress on the IVF treatment outcome is unclear. This study aimed to examine the effects of anxiety, depression, and stress symptoms before IVF treatment on the clinical pregnancy rate, controlling for known confounders.

**Results:**

In total, 142 women undergoing IVF treatment participated in this prospective study. The clinical pregnancy rate was 26.8% in this study. Controlling for age, infertility duration, and cause of infertility, there were no relationship between IVF outcome and anxiety (relative risk (RR) = 1.00; 95% CI 0.91–1.09), depression (RR = 0.96; 95% CI 0.88–1.05), and stress (RR = 1.01; 95% CI 0.96–1.07) symptoms. High woman’s age and women with both cause of infertility were independent predictors of IVF clinical pregnancy rate. In sum, we found that anxiety, depression, and stress symptoms were not associated with the IVF clinical pregnancy rate.

## Introduction

Infertility is defined by “the failure to establish a clinical pregnancy after 12 months of regular, unprotected sexual intercourse or due to an impairment of a person’s capacity to reproduce either as an individual or with his/her partner.” [1], and affects about 9% of reproductive-age couples [2]. Infertility is one of the greatest stressors in life that results in psychological distress. Anxiety, depression, and stress symptoms are the most frequently occurring psychological disorders among infertile patients [3–5]. Regarding gender, it is widely accepted that women are more affected than men by infertility problem, particularly in developing countries. For example, in some studies conducted among infertile couples, women were more likely to have psychological disorders and impaired quality of life than men [6, 7].

The use of assisted reproductive technology (ART) treatment, especially in vitro fertilization (IVF), has grown rapidly in the past two decades. Women undergoing ART treatment, are often anxious and depressed because of their infertility and the uncertainties of the treatment with which they have to deal [8]. Although some studies report that the outcome of ART treatment may be dependent on psychological factors, such as a women’s distress level before and during ART treatment [9–11], other studies find no association on this topic [12, 13]. These contradicting results may have been caused by the small samples, non-standardized psychological tests, the design of the study, or population characteristics. Furthermore, except for women’s age, other potential predictors such as infertility duration and cause of infertility often have not been controlled for. The objective of this study was to examine the effects of stress, anxiety and depression symptoms on IVF results, controlling for known confounders.

## Main text

### Methods

#### Participants and study design

This prospective study conducted on 142 women undergoing IVF treatment at Royan Infertility Clinic which is a referral center for infertility in Tehran, Iran [14]. We collected the data from February to March 2017. To be eligible for the study the women had to meet the following criteria: (a) experiencing couple infertility, (b) age over 18 years; (c) ability to read, write, and comprehend Persian. Ethic approval to carry out this study was given by the Ethics Committee of Royan Institute, Tehran, Iran. Each woman gave written consent prior to participating in the study.

#### Measures

##### Hospital Anxiety and Depression Scale (HADS)

The HADS is a widely used self-administered instrument that measures both anxiety (HADS-A, 7 items) and depression (HADS-D, 7 items) symptoms [15]. Each item is scored on a 4-point Likert scale ranging from 0 to 3. Both subscale scores range from 0 to 21, with higher scores indicating greater symptoms of anxiety and depression. The Persian language version of the HADS has shown adequate psychometric properties among infertile patients [16] and widely used in this population [17, 18]. In this study, the Cronbach’s alpha coefficients for HADS-A and HADS-D subscales were 0.846 and 0.755, respectively.

##### Perceived Stress Scale-10 (PSS-10)

The PSS-10 is a widely used self-administered instrument that measures “the degree to which situations in one’s life over the last month are appraised as unpredictable, uncontrollable and overloading” [19]. Respondents rate items on a 5-point Likert-type scale, ranging from 0 (never) to 4 (very often). The total PSS-10 score can range from 0 to 40, with higher scores meaning greater stress [19]. The Persian language version of PSS-10 has shown adequate psychometric properties among infertile patients [20] and adults with asthma [21]. In this study, the Cronbach’s alpha coefficient for the PSS-10 was 0.850.

#### Data analysis

Regarding the type of the study (prospective study) and main outcome (pregnancy), negative binomial regression model was used to examine the effect of psychological distress on clinical pregnancy. Therefore, we calculated relative risks (RR) and adjusted relative risks (RR_Adj_) with 95% confidence intervals (CI). The women’s age, duration of infertility and cause of infertility were used in adjusted analysis. In addition, comparison between pregnant and nonpregnant women on psychological distress scores was done using independent t test. Data analysis was done with IBM SPSS Statistics for Windows, Version 24.0 (IBM Corp., Armonk, NY, USA). All statistical tests were two-tailed and level of significance was set at 0.05.

### Results

In total, 142 women agreed to take part in this research and filled out the questionnaires completely. The mean age of women was 32.04 (SD = 5.52) years and infertility duration was 7.04 (SD = 4.36) years. Of these women, 35.2% had university education and 16.2% were employed. The causes of infertility were as follows: male factor, 47.2%; female factor, 23.9%; both, 12.7%; and unknown, 16.2%.

The clinical pregnancy rate was 26.8% in this study. As seen in Table 1, there were no significant difference between pregnant and nonpregnant women with respect to anxiety (P = 0.974), depression (P = 0.599), and stress (P = 0.498) scores.

**Table 1:** Comparison between pregnant and nonpregnant women on anxiety, depression, and stress

	Pregnant	Nonpregnant	P
Anxiety	7.42 (4.47)	7.39 (4.25)	0.974
Depression	5.39 (3.37)	5.76 (3.76)	0.599
Stress	17.76 (6.72)	16.87 (7.07)	0.498

Values are presented as mean (SD)

According to simple analysis, there was no relationship between IVF clinical pregnancy rate and anxiety (RR = 1.00; 95% CI 0.94–1.07), depression (RR = 0.98; 95% CI 0.91–1.06), and stress (RR = 1.01; 95% CI 0.98–1.05) symptoms. Among known confounders, only high mother’s age was associated with a decreased rate of IVF results (RR = 0.94; 95% CI 0.91–0.98) (Table 2).

**Table 2:** Simple and multiple analyses of psychological and biological factors predicting the IVF pregnancy rate

	Simple analysis		Multiple analysis^a^	
	RR_Crude_ (95% CI)	P	RR _Adj_ (95% CI)	P
Age (years)	0.94 (0.91–0.98)	0.002	0.94 (0.90–0.98)	0.004
Infertility duration (years)	0.96 (0.91–1.02)	0.169	0.98 (0.92–1.04)	0.435
Cause of infertility				
Female factor	1	–	1	–
Male factor	0.81 (0.35–1.87)	0.616	0.83 (0.38–1.82)	0.639
Both	0.34 (0.09–1.38)	0.133	0.27 (0.07–1.00)	0.050
Unknown	0.88 (0.47–1.63)	0.676	0.69 (0.36–1.34)	0.278
Anxiety	1.00 (0.94–1.07)	0.974	1.00 (0.91–1.09)	0.917
Depression	0.98 (0.91–1.06)	0.580	0.96 (0.88–1.05)	0.398
Stress	1.01 (0.98–1.05)	0.485	1.01 (0.96–1.07)	0.712

RR: relative risk, CI: confidence interval

^a^Adjusted for age, infertility duration, and cause of infertility

Controlling for age, infertility duration, and cause of infertility, there were also no association of anxiety, depression, and stress symptoms with IVF results (RR_Adj_ = 1.00, 95% CI 0.91–1.09; RR_Adj_ = 0.96, 95% CI 0.88–1.05; RR_Adj_ = 1.01, 95% CI 0.96–1.07, respectively). According to multiple analysis, women with both cause of infertility were less likely to have a clinical pregnancy than women with female infertility (RR_Adj_ = 0.27; 95% CI 0.07–1.00).

### Discussion

In this prospective study, we evaluated the effects of stress, anxiety and depression symptoms on the clinical pregnancy rate for women having their first IVF treatment. Both simple and multiple analyses indicated that stress symptom had no influence on the outcome of IVF treatment, which is consistent with some studies [12, 13], but not with others [9–11]. Consistent with previous research on anxiety and depression symptoms before and during first IVF treatment, we did not find effects of anxiety and depression symptoms on the clinical pregnancy rate [12, 13, 22]. However, other studies indicate that psychological distress are related to IVF outcomes [9–11, 23]. On the other hand, infertile women had high level of depression and anxiety symptoms and low quality of life after unsuccessful ART treatment [3, 5, 18, 24]. We therefore do recommend a holistic approach, including psychosocial interventions and support to reduce the level of psychological distress in these women.

In accordance with previous research [13, 25], women’s age showed a significant impact on clinical pregnancy rate. Furthermore, in comparison with women who had female infertility, women with both cause of infertility had a significantly increased risk of pregnancy rate. Infertility duration did not have an effect on the outcome of IVF treatment. This factor has shown an influence on clinical pregnancy in two large prospective studies [25, 26].

In sum, we found that anxiety, depression, and stress symptoms were not associated with the IVF clinical pregnancy rates. Although the study sample was small, the small confidence intervals obtained from the multiple regression analysis implicate accurate results.

## Limitations

The present study has several limitations that should be noted when interpreting the findings. First, because the study was conducted in only one center, the generalizability of the findings may be limited. Second, the study sample was relatively small. Third, we are unable to examine the relationship between these psychological distress and live birth and miscarriage.

## Data Availability

The datasets used and/or analyzed during the current study available from the corresponding author on reasonable request.

## Abbreviations

ART: assisted reproductive technology
IVF: in vitro fertilization
RR: relative risk
CI: confidence interval
HADS: hospital anxiety and depression scale
PSS-4: Perceived Stress Scale-4 Item
